# Identifying People Living With or Those at Risk for HIV in a Nationally Sampled Electronic Health Record Repository Called the National Clinical Cohort Collaborative: Computational Phenotyping Study

**DOI:** 10.2196/68143

**Published:** 2025-07-11

**Authors:** Eric Hurwitz, Cara D Varley, A Jerrod Anzolone, Vithal Madhira, Amy L Olex, Jing Sun, Dimple Vaidya, Nada Fadul, Jessica Y Islam, Lesley E Jackson, Kenneth J Wilkins, Zachary Butzin-Dozier, Dongmei Li, Sandra E Safo, Julie A McMurry, Pooja Maheria, Tommy Williams, Shukri A Hassan, Melissa A Haendel, Rena C Patel, The National Clinical Cohort Collaborative (N3C) Consortium

**Affiliations:** 1 Department of Genetics University of North Carolina at Chapel Hill Chapel Hill, NC United States; 2 School of Medicine Oregon Health & Science University Portland, OR United States; 3 Department of Biostatistics University of Nebraska Medical Center Omaha, NE United States; 4 Palila Software Reno, NV United States; 5 Wright Center for Clinical and Translational Research Virginia Commonwealth University Richmond, VA United States; 6 Department of Epidemiology Johns Hopkins Bloomberg School of Public Health Baltimore, MD United States; 7 Department of Medicine University of Washington Seattle, WA United States; 8 Department of Internal Medicine University of Nebraska Medical Center Omaha, NE United States; 9 Department of Cancer Epidemiology H. Lee Moffitt Cancer Center and Research Institute Tampa, FL United States; 10 Department of Oncologic Sciences University of South Florida Tampa, FL United States; 11 Department of Medicine University of Alabama at Birmingham Birmingham, AL United States; 12 Biostatistics Program Office of the Director National Institute of Diabetes and Digestive and Kidney Diseases National Institutes of Health Bethesda, MD United States; 13 Division of Biostatistics University of California Berkeley School of Public Health Berkeley, CA United States; 14 Department of Clinical and Translational Research University of Rochester Medical Center Rochester, NY United States; 15 Department of Biostatistics School of Public Health University of Minnesota Minneapolis, MN United States; 16 See Acknowledgments

**Keywords:** HIV, electronic health records phenotype, epidemiologic methods, COVID-19, preexposure prophylaxis, postexposure prophylaxis

## Abstract

**Background:**

Electronic health records (EHRs) provide valuable insights to address clinical and epidemiological research concerning HIV, including the disproportionate impact of the COVID-19 pandemic on people living with HIV. To identify this population, most studies using EHR or claims databases start with diagnostic codes, which can result in misclassification without further refinement using drug or laboratory data. Furthermore, given that antiretrovirals now have indications for both HIV and COVID-19 (ie, ritonavir in nirmatrelvir/ritonavir), new phenotyping methods are needed to better capture people living with HIV. Therefore, we created a generalizable and innovative method to robustly identify people living with HIV, preexposure prophylaxis (PrEP) users, postexposure prophylaxis (PEP) users, and people not living with HIV using granular clinical data after the emergence of COVID-19.

**Objective:**

The primary aim of this study was to use computational phenotyping in EHR data to identify people living with HIV (cohort 1), PrEP users (cohort 2), PEP users (cohort 3), or “none of the above” (people not living with HIV; cohort 4) and describe COVID-19–related characteristics among these cohorts.

**Methods:**

We used diagnostic and laboratory measurements and drug concepts in the National Clinical Cohort Collaborative to create a computational phenotype for the 4 cohorts with confidence levels. For robustness, we conducted a randomly sampled, blinded clinician annotation to assess precision. We calculated the distribution of demographics, comorbidities, and COVID-19 variables among the 4 cohorts.

**Results:**

We identified 132,664 people living with HIV with a high level of confidence, 36,088 PrEP users, 4120 PEP users, and 20,639,675 people not living with HIV. Most people living with HIV were identified by a combination of medical conditions, laboratory measurements, and drug exposures (74,809/132,664, 56.4%), followed by laboratory measurements and drug exposures (15,241/132,664, 11.5%) and then by medical conditions and drug exposures (14,595/132,664, 11%). A higher proportion of people living with HIV experienced COVID-19–related hospitalization (4650,132,664, 3.5%) or mortality (828/132,664, 0.6%) and all-cause mortality (2083/132,664, 1.6%) compared to other cohorts.

**Conclusions:**

Using an extensive phenotyping algorithm leveraging granular data in an EHR repository, we have identified people living with HIV, people not living with HIV, PrEP users, and PEP users. Our findings offer transferable lessons to optimize future EHR phenotyping for these cohorts.

## Introduction

### Background

In the United States, there are an estimated 1.2 million people living with HIV, with 32,100 new cases diagnosed in 2021 [1]. The COVID-19 pandemic has disproportionately impacted people living with HIV, who have a 4-fold higher likelihood of contracting COVID-19 and experiencing severe outcomes, such as increased disease severity, higher hospitalization rates, and elevated mortality [2-4]. Initially, the COVID-19 pandemic introduced challenges to optimal HIV management, including a substantial reduction in HIV testing, resulting in underdiagnosis and barriers to necessary treatment [5-8]. Beyond its effects on people living with HIV, the COVID-19 pandemic also disrupted preexposure prophylaxis (PrEP) use and availability, potentially increasing the vulnerability of those at risk of acquiring HIV [9]. For example, PrEP users experienced a 6% to 11.5% decline in medication coverage early in the pandemic [5,9,10]. Disparities related to social determinants of health that have historically affected minoritized (ie, racial, ethnic, gender, rural, and socioeconomic) communities with HIV became more apparent since the onset of the COVID-19 pandemic [11-15]. The pronounced impact of COVID-19 on people living with HIV and those at risk for HIV underscores the urgent need for focused research within this population for better preparedness for the next public health crisis that may affect an already vulnerable population.

Electronic health records (EHRs) provide valuable insights to address specific clinical and epidemiological research concerning HIV, including the disproportionate impact of the COVID-19 pandemic on this population [2,13-15]. The National Clinical Cohort Collaborative (N3C) is a nationally sampled EHR repository in the United States with granular, individual-level clinical data from >98 data partner sites and houses >32 billion rows of data for >22 million individuals [16]. These rich EHR data have allowed investigation of questions related to the COVID-19 pandemic and HIV, including identification of an elevated risk of poor COVID-19 outcomes in people living with HIV with a low cluster of differentiation 4 (CD4) count (<200 cells/µL) and racial disparities with COVID-19 positivity among people living with HIV, in addition to both individual and area-level social determinants of health with COVID-19 outcomes [2,13-15]. The N3C Enclave not only provides a large sample size for answering research questions but also contains granular individual-level information, such as social determinants of health [17], visit and prescription frequencies, and geographic information to better identify health disparities during the COVID-19 pandemic [18,19].

Nonetheless, identifying potential people living with HIV, PrEP users, or postexposure prophylaxis (PEP) users in any EHR dataset necessitates indirectly inferring data from source information. Most studies using EHR or claims databases start with diagnostic codes, which can result in misclassification without further refinement using drug or laboratory data [20-22]. While these previous studies have performed computational phenotyping of HIV on smaller cohorts, with data or validation often limited to a single institution or health system, one study [23], conducted with All of Us data, has included additional complexity such as self-reporting of medical conditions, which is not always feasible with large datasets [22-26]. While each approach has strengths, they also suffer from limitations, such as (1) variation in the availability of EHR data among individuals that potentially leads to differential misclassification, (2) use of nonstandardized codes specific to a single health system preventing interoperability across health systems, (3) inability to distinguish between individuals with high- or low-confidence designations, (4) additional misclassification with increased uptake of PrEP or PEP, and (5) the use of antiretrovirals that now have indications for both HIV and COVID-19 treatment (ie, ritonavir in nirmatrelvir/ritonavir) resulting in misclassification.

### Objectives

Our objective was to use an innovative approach after the emergence of COVID-19 to robustly identify people living with HIV, PrEP users, PEP users, and people not living with HIV using granular clinical data in N3C, augmented by clinician annotation without chart abstraction of source data. We used the standardized Observational Medical Outcomes Partnership (OMOP) Common Data Model to create 4 cohorts or “phenotypes,” including people living with HIV (cohort 1), PrEP users (cohort 2), PEP users (cohort 3), and people not living with HIV (cohort 4), based on medical conditions, laboratory measurements, and drug data available in N3C. We conducted a clinician-curated annotation to reduce and describe misclassification. In addition, we introduced confidence levels to classify individuals into categories of low, medium, or high confidence in their status as people living with HIV, allowing researchers to tailor the cohorts to their specific research needs. These cohorts will serve as a framework for future research to address questions related to HIV within N3C, and our methods inform more generalizable approaches to phenotyping HIV in EHR data.

## Methods

### Study Population

The N3C Enclave contains EHR data from clinical sites and represents the largest limited dataset of COVID-19 cases and controls in the United States. N3C contains harmonized EHR data for individuals who tested positive and negative for COVID-19 from January 1, 2020, to present through routine weekly updates to incorporate near real-time clinical encounters and procedures, in addition to historical data dating back to January 1, 2018. Data include individuals who tested positive for COVID-19 matched with 2 controls who tested negative for COVID-19 based on a maximum of 4 sociodemographic variables (age, sex, race, and ethnicity) whenever available by data partner site. Our cohorts of people living with HIV, PrEP users, PEP users, and people not living with HIV were defined based on the N3C Enclave data release as of November 2, 2023 (version 148; from 93 data partner sites) using a limited dataset.

### Procedures

#### HIV Phenotyping Overview Scheme

Our cohorts were identified in the N3C Enclave through the use of OMOP concepts, where concept IDs, concept set names, and our phenotyping pipeline can be searched in our public-facing GitHub repository for transferable methods in and out of N3C [27]. These standardized concepts correspond to three major sources of data: (1) Systematized Medical Nomenclature for Medicine Clinical Terminology (SNOMED CT) codes and *International Classification of Diseases, Tenth Revision* (*ICD-10*) mapped to SNOMED CT codes for HIV diagnosis from the HIV-related condition occurrence table; (2) Logical Observation Identifiers Names and Codes for HIV measurements from the measurement table; and (3) HIV medication exposures from the drug exposure table in RxNorm [28]. The selection of OMOP concepts across these 3 domains was carried out using the Observational Health Data Sciences and Informatics Atlas tool [29,30], in collaboration with HIV clinicians, to identify and include all relevant HIV-related OMOP concept IDs.

**Figure 1:**
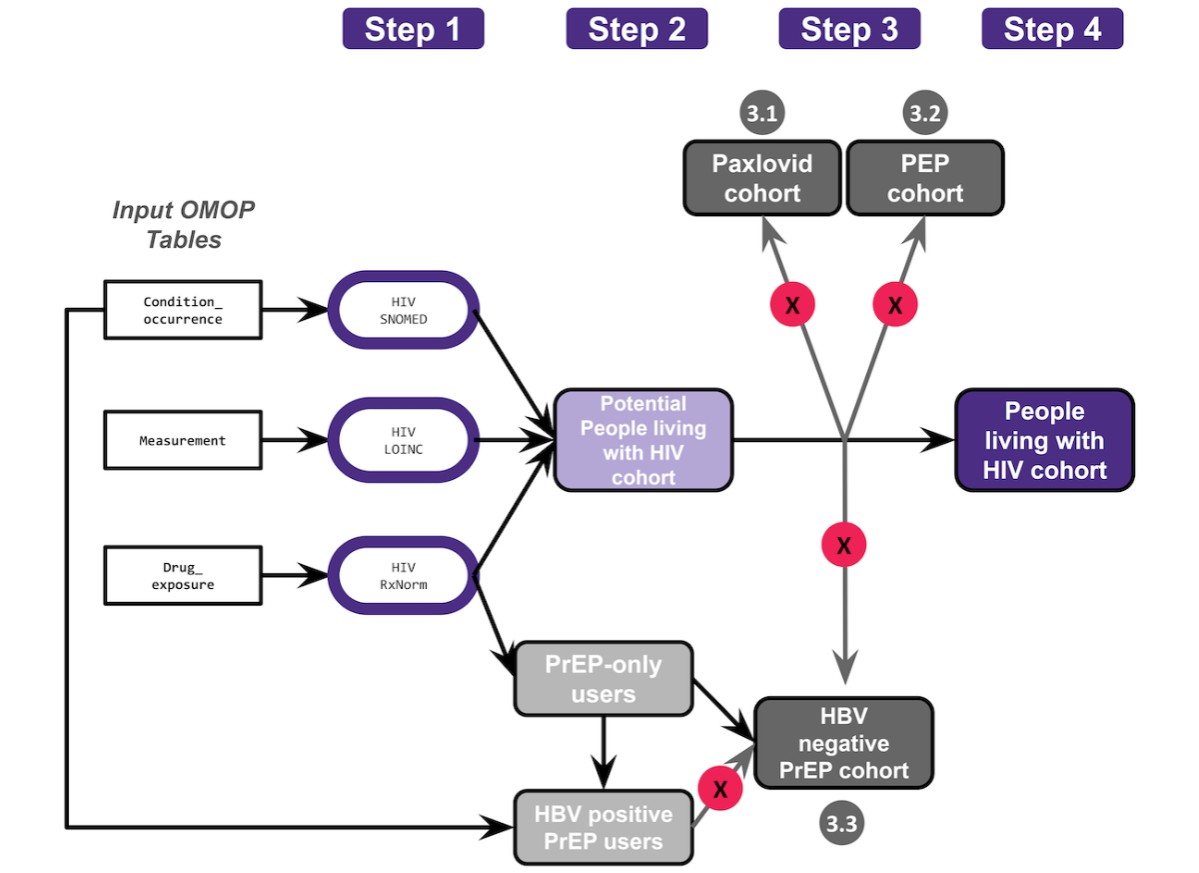
A schematic detailing the process of creating our people living with HIV cohort. Individuals were included in the HIV cohort by a combination of medical conditions, laboratory measurements, or drug exposures (step 1) to create a potential cohort of people living with HIV (step 2). Individuals taking nirmatrelvir/ritonavir (step 3.1), postexposure prophylaxis (PEP) users (step 3.2), and pre-exposure prophylaxis (PrEP) users (step 3.3) were excluded from the intermediate cohort (step 3), which is depicted by “X” in the red circle, to create a final cohort of people living with HIV (step 4). HBV: hepatitis B virus; LOINC: Logical Observation Identifiers Names and Codes; OMOP: Observational Medical Outcomes Partnership; SNOMED: Systematized Medical Nomenclature for Medicine.

Conceptually, our approach identified possible people living with HIV, followed by subtyping PrEP and PEP users and removing people using HIV drugs for only chronic hepatitis B virus (HBV) or COVID-19 infection (Figure 1). From the potential cohort of people living with HIV, using the table of individuals prescribed HIV-related drugs, we phenotyped PrEP users using concept IDs in the *(immunosuppressed or compromised) PrEP concept set* and excluding individuals with an HIV diagnostic code or positive laboratory measurement (eg, antibody and antigen, viral load [VL] ≥50 copies/mL, or CD4 ≤200 cells/µL) [27]. The final cohort of PrEP users was created by excluding individuals with HBV infection to avoid potential misclassification (as tenofovir disoproxil fumarate [TDF] alone is often used for HBV treatment and avoided for PrEP to reduce the risk of acute hepatitis flare upon discontinuation of TDF for PrEP). From the potential cohort of people living with HIV, identified earlier by drug exposures (after excluding PrEP users), PEP users were identified using the concepts in the *PEP concept set* [27]. Individuals with ritonavir-only exposure (ie, those with no other antiretroviral exposure for HIV treatment, no medical conditions, and no laboratory measurements), were categorized as “uncertain classification,” given the overlap of ritonavir use for COVID-19 treatment in nirmatrelvir/ritonavir using concepts listed in *Ritonavir concept set* and *Paxlovid concept set* [27]. In addition to our “ritonavir only” group, we identified those with uncertain classification status for whom, despite our best efforts, we could not adjudicate with precision which cohort they belonged to and therefore excluded these individuals from all cohorts. For instance, some individuals had repeat HIV VL testing without any results or other HIV-related laboratory measurements, drug exposures, or medical conditions, where the repeat testing could represent monitoring not only HIV treatment but also HIV screening. We considered this uncertain classification to be of low confidence, with high concern for misclassification. Given the risk of additional misclassification that assigning such individuals to one of the 4 specific cohorts would cause, we chose to remove them from the 4 specific cohorts altogether. Thus, the final cohort of people living with HIV was created by including individuals positive for HIV by medical conditions, laboratory measurements, or drug exposures and excluding any individuals listed in the final PrEP, PEP, and “ritonavir-only” cohorts (Figure 1).

#### Data Preprocessing

Laboratory measurements, such as absolute CD4 cell count and percentage and VL measures, are crucial to research among people living with HIV. To extract meaningful data related to CD4 and VL measurements, we first identified concept IDs for CD4 and VL measurements seen in the *CD4 Cell Count (Absolute)* and the *HIV Viral Load (version 3) concept sets* [27]. CD4 and VL phenotyping were performed on all individuals in N3C using the measurement table.

#### Applying Levels of Confidence to the Cohort of People Living With HIV

To determine, with varying levels of confidence, who within the cohort were people living with HIV, we assigned a confidence level between 1 and 3 (with 1 indicating the highest confidence) per individual to reflect the degree of certainty in their status as someone living with HIV. Briefly, confidence level 1 included individuals with positive HIV laboratory results; confidence level 2 comprised individuals identified through a combination of medical conditions, the presence of laboratory measurements, and HIV medication exposures but without available laboratory results; and confidence level 3 consisted of individuals identified solely based on HIV drug exposures or only 1 record of an HIV diagnosis. More details are provided in the applying levels of confidence to the cohort of people living with HIV section in Multimedia Appendix 1 [16,31-33].

#### PrEP Phenotyping

In order to identify PrEP users, we first identified those using PrEP drugs (emtricitabine and tenofovir) from the possible HIV cohort who were identified by drug exposures only (ie, excluding individuals with an HIV diagnostic code or positive laboratory measurement, such as antibody and antigen, VL ≥50 copies/mL, or CD4 ≤200 cells/µL). Due to low numbers, cabotegravir for PrEP was not included. Among these, as noted earlier, individuals with likely chronic HBV infection were excluded based on concept IDs listed in the *HBV concept set* [27]. Further details about PrEP phenotyping and assigning confidence levels for PrEP users are provided in Multimedia Appendix 1.

#### PEP Phenotyping

PEP phenotyping was performed using the potential cohort of people living with HIV identified by drug exposures after excluding those in the PrEP cohort. We then filtered individuals based on the following criteria: (1) had a record of emtricitabine/TDF and dolutegravir or emtricitabine/TDF and raltegravir use on the same date, (2) had a supply of <30 days (or the supply information was null), and (3) only had one row of data (indicating they were not receiving an HIV treatment regimen repeatedly, which would otherwise be more indicative of an HIV-infection–related treatment). Given that our phenotyping approach was dependent on accounting for all data available over time, it was not possible to distinguish, with confidence, PEP users who later acquired HIV. Concepts used in this workflow are available in the *PEP concept set* [27]. Given the overall small size of the PEP cohort, we chose not to pursue confidence level typing of this group.

#### Ritonavir-Only Phenotyping

We recognized that several individuals may have been prescribed ritonavir as part of nirmatrelvir/ritonavir (ie, Paxlovid) or another off-label regimen as a COVID-19 therapeutic. Therefore, those entering our cohort of people living with HIV by drug exposures only and who had only been prescribed ritonavir (without any other antiretrovirals used for HIV treatment documented) after January 1, 2020, were categorized separately for further evaluation using concepts described earlier [27]. We then identified those taking ritonavir as part of nirmatrelvir/ritonavir via concepts and drug source values. Individuals who only used ritonavir as part of nirmatrelvir/ritonavir were subsequently excluded. Given that there were no other laboratory tests, medical conditions, or drug exposures consistent with HIV, we suspected this group reflected people not living with HIV; however, due to the uncertainty of the indication for the ritonavir prescription, we categorized them in an uncertain classification.

#### Clinician Annotation Activity

To ensure the robustness of our cohorts of people living with HIV, PrEP users, and PEP users, clinicians experienced in treating HIV conducted a blinded annotation activity. Briefly, clinician annotation involving 120 randomly selected individuals (n=90, 75% people living with HIV; n=10, 8.3% PrEP users; n=10, 8.3% PEP users; and n=10, 8.3% individuals not living with HIV) was performed. Three clinicians with experience of treating HIV, blinded to the study procedures, reviewed row-level HIV EHR data (including medical conditions, laboratory measurements, and drug exposures) and classified each individual as person living with HIV, PrEP user, PEP user, or person not living with HIV based on the available data for each individual. For each clinician, we generated a confusion matrix and calculated sensitivity, specificity, positive predictive value, negative predictive value, precision, recall, and *F*_1_-score. Multimedia Appendix 1 provides further details about this process.

#### Demographics and Comorbidity Definitions

Demographics and comorbidities of individuals were defined using standardized N3C-wide definitions. The set of comorbidities selected included those in the Charlson Comorbidity Index. Concept sets were created for each medical condition listed using the primary conditions listed in the SNOMED CT hierarchy, including all descendants. Specific details for each concept set can be found in the N3C concept set browser and are described in the data dictionary [16,34].

#### COVID-19 Outcome Definitions

The COVID-19 severity and outcomes for individuals were defined using N3C-wide definitions. In this analysis, COVID-19 positivity was defined by (1) a set of a priori–defined SARS-CoV-2 laboratory tests (that included polymerase chain reaction or antigen positivity but not antibody positivity) or (2) a “strong positive” diagnostic code (this cohort code is available on GitHub) [35,36].

### Ethical Considerations

Direct patient consent was not obtained for this repository of deidentified data per N3C policies. The N3C received a waiver of consent from the National Institutes of Health (NIH) Institutional Review Board (IRB), and NIH takes care to ensure the highest privacy and security requirements are met and adhered to for housing and protecting these data in the NIH-managed N3C Enclave. More details can be found in N3C resources [37].

The N3C Enclave is approved through the NIH IRB. Each individual data partner site maintains its own IRB-approved data transfer agreement or joins under a Johns Hopkins University Reliance Protocol (IRB00249128). Each investigator accessing the N3C Enclave receives local IRB approval from their respective institutions. The N3C Data Access Committee approved this project (RP-CA3365).

## Results

### Identifying People Living With HIV, PrEP Users, and PEP Users in N3C

**Figure 2:**
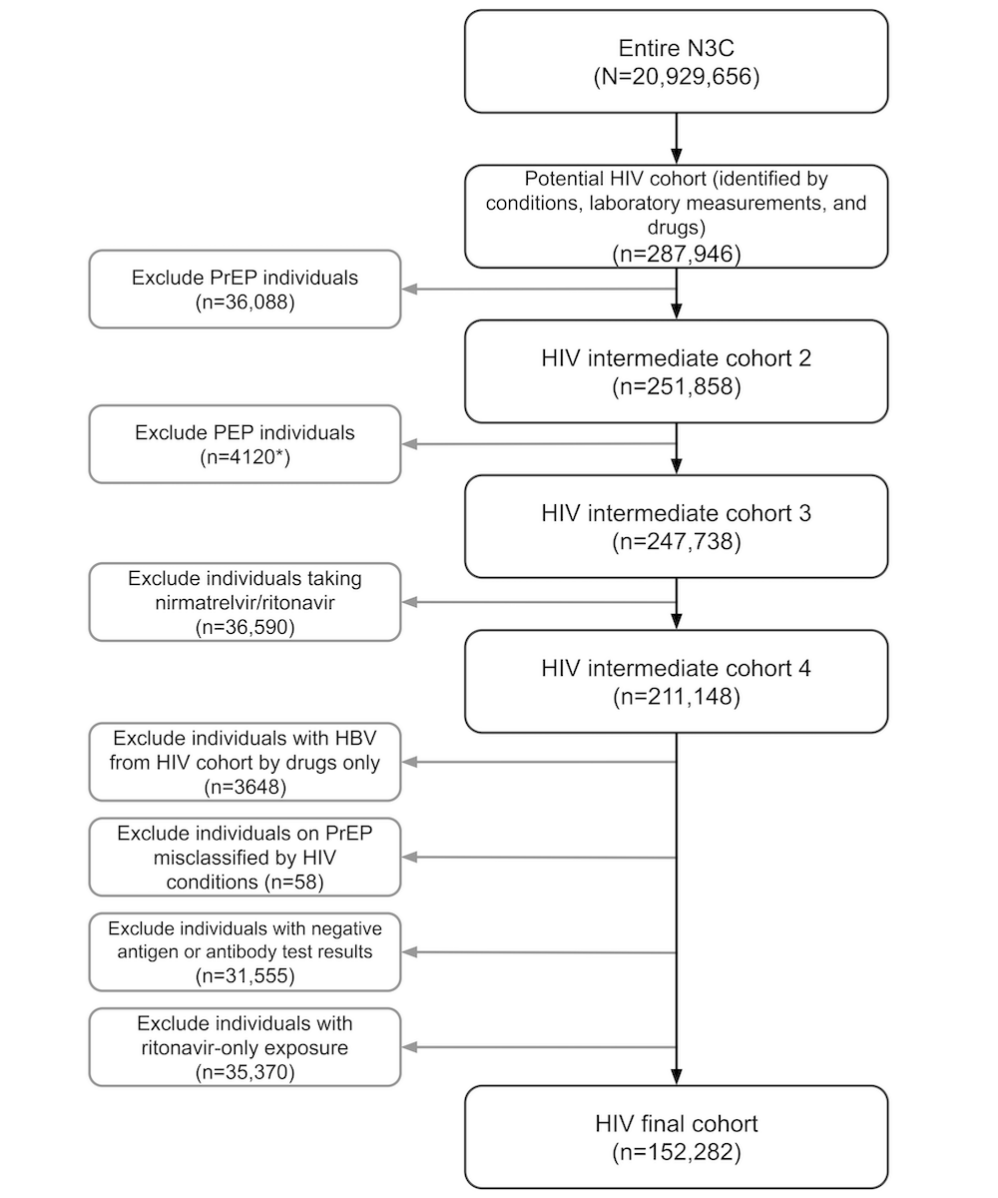
Flow diagram describing the inclusion and exclusion criteria of the HIV cohort. *Total number of postexposure prophylaxis (PEP) users is presented as an estimate due to National Clinical Cohort Collaborative (N3C) governance rules to obfuscate counts <20. HBV: hepatitis B virus; PrEP: pre-exposure prophylaxis.

**Figure 3:**
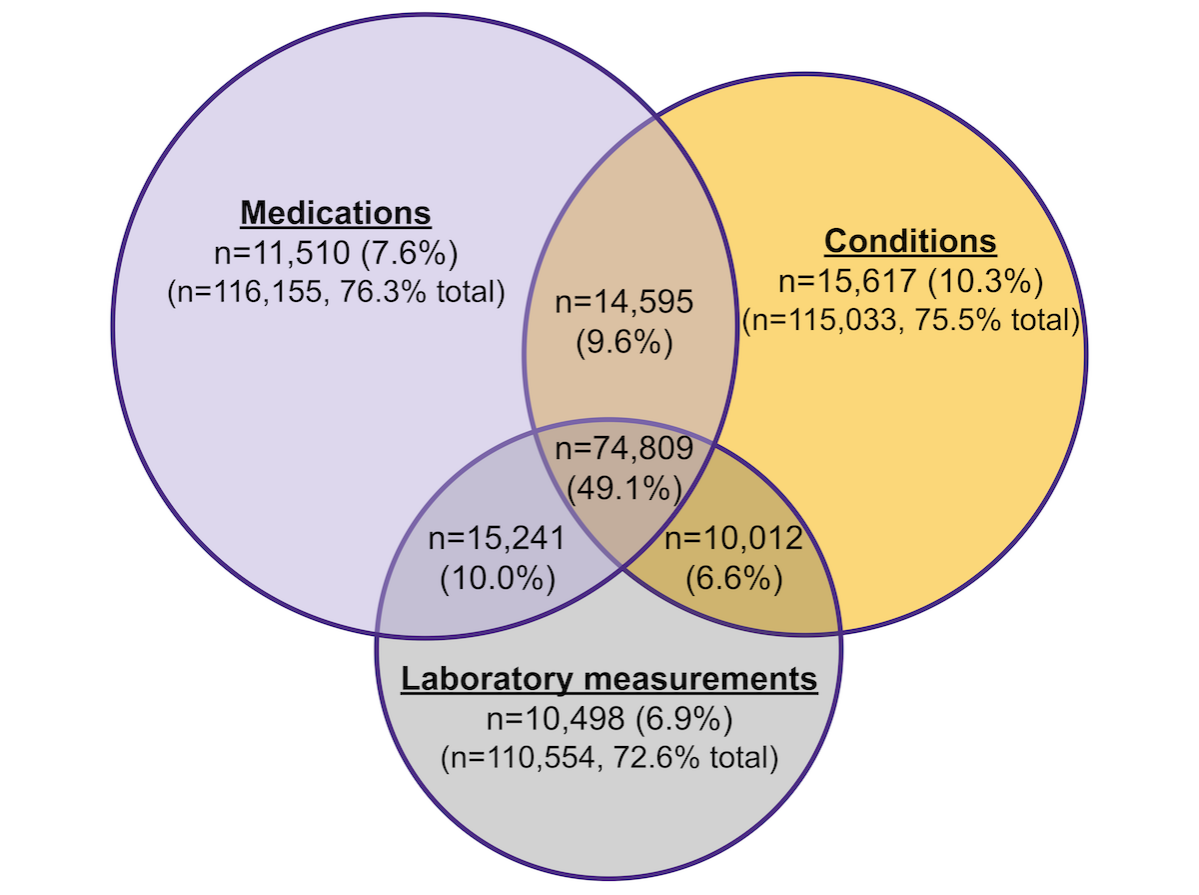
A Venn diagram displaying the number of individuals in the HIV cohort identified by medical conditions, laboratory measurements, and drug exposures (n=152,282). Top numbers represents the count (and percentage) of individuals in specific categories (eg, 14,595 individuals identified by the combination of medications and medical conditions), while bottom numbers display the number of individuals broadly identified by medical conditions, laboratory measurements, and medications (eg, 116,155 individuals in the HIV cohort have an HIV-related condition).

Using a computational phenotype through a combination of medical conditions, laboratory measurements, and medication data, we identified 152,282 (0.7%) individuals as people living with HIV from a total population of 20,928,656 (Figure 2). Among these cases, many were recognized through the combination of medical conditions, laboratory measurements, and drug exposures (n=74,809, 49.1%), followed by medical conditions alone (n=15,617, 10.3%), and then by laboratory measurements and drug exposures (n=15,241, 10%; Figure 3). When categorizing people living with HIV based on confidence levels, we found that most individuals fell into confidence level 1 (n=108,068, 71%), followed by level 2 (n=24,596, 16.2%), and then level 3 (n=19,618, 12.9%; Table 1). Our phenotyping process also identified 36,088 PrEP users (after excluding 303 individuals for HBV monoinfection), 4120 PEP users (the total number of PEP users is presented as an estimate due to N3C governance rules to obfuscate counts <20), and 96,491 individuals with uncertain classification (Table 2). In terms of our confidence levels for each PrEP user, most individuals were categorized as level 1 (20,742/36,088, 57.5%), followed by level 3 (11,560/36,088, 32%) and then level 2 (3786/36,088, 10.5%; Table 3). We did observe a small number of individuals from the people living with HIV cohort (24/152,282, 0%) with possible PrEP exposure before a diagnosis of HIV.

**Table 1:** Counts and percentages of people living with HIV in each confidence level by criterion (n=152,282).

Confidence level		Count, n (%)
**Level 1** **total**		108,068 (71)
	Criterion 1	41,923 (27.5)
	Criterion 2	28,512 (18.7)
	Criterion 3	1379 (0.9)
	Criterion 4	5606 (3.7)
	Criterion 5	18,499 (12.1)
	Criterion 6	12,495 (8.2)
**Level 2 total**		24,596 (16.2)
	Criterion 7	264 (0.2)
	Criterion 8	13,061 (8.6)
	Criterion 9	7509 (4.9)
**Level 3 total**		19,618 (12.9)
	Criterion 10	7078 (4.6)
	Criterion 11	4432 (2.9)
	Criterion 12	8108 (5.3)

**Table 2:** Counts and percentages of individuals in each cohort (N=20,928,656)^a^.

Cohort	Count, n (%)
People living with HIV (confidence levels 1-3)	152,282 (0.7)
People not living with HIV	20,639,675 (98.6)
PrEP^b^ users	36,088 (0.2)
PEP^c^ users	4120 (0)
Uncertain classification	96,491 (0.5)

^a^Count for the entire National Clinical Cohort Collaborative.

^b^PrEP: preexposure prophylaxis.

^c^PEP: postexposure prophylaxis.

**Table 3:** Counts and percentages of preexposure prophylaxis (PrEP) users in each confidence level (n=36,088).

Confidence level	Count, n (%)
Level 1	20,742 (57.5)
Level 2	3786 (10.5)
Level 3	11,560 (32)

### Verification of Cohort of People Living With HIV, PrEP Users, and PEP Users via a Clinician Annotation Activity

To enhance the reliability of our methods, we conducted a clinician annotation process aimed at assessing the accuracy of our phenotyping methods for people living with HIV, PrEP users, and PEP users. Our results demonstrated excellent sensitivity (range 0.90-1), specificity (range 0.97-1), positive predictive value (range 0.77-1), negative predictive value (range 0.88-1), precision (range 0.77-1), recall (range 0.90-1), and *F*_1_-score (range 0.87-1; Multimedia Appendix 2). Similarly, the κ values exhibited high levels of interrater reliability (range 0.91-0.98; Multimedia Appendix 2).

### Characteristics of People Living With HIV, PrEP Users, and PEP Users in N3C

To obtain a more comprehensive understanding of the individuals within our various cohorts, we described their sociodemographic characteristics. Within the cohort of people living with HIV in the highest 2 confidence levels (132,664/152,282, 87.1% of all potential people living with HIV identified), the highest proportion of non-Hispanic Black individuals is in the people living with HIV cohort (49,769/132,664, 37.5%), followed by PEP users (784/4120, 19%), people not living with HIV (2,909,443/20,639,675, 14.1%), and finally among PrEP users (4534/36,088, 12.6%; Table 4). Notably, PrEP users contained the highest proportion of Hispanic or Latinx individuals (5829/36,088, 16.2%) of any race and ethnicity compared to other cohorts (people living with HIV: 20,925/132,664, 15.8%; PEP users: 403/4120, 9.8%; people not living with HIV: 2,628,308/20,639,675, 12.7%; Table 4). Male individuals constituted a higher proportion of people living with HIV (90,496/132,664, 68.2%) and PrEP users (31,722/36,088, 87.9%), which was notably higher than the proportion of male individuals among PEP cohorts (1399/4120, 34%) and people not living with HIV (9,044,853/20,639,675, 43.8%; Table 4). The median ages of people living with HIV, PrEP users, PEP users, and people not living with HIV were 51 (IQR 38-61), 36 (IQR 30-46), 35 (IQR 28-44), and 44 (IQR 26-63) years, respectively, demonstrating that PrEP and PEP users were generally younger than people living with HIV and people living without HIV (Table 4). We also examined comorbidities among people living with HIV compared to other cohorts and found that people living with HIV experienced chronic lung disease, liver disease, hypertension, kidney disease, and diabetes more often compared to PrEP users, PEP users, and people not living with HIV (Table 4).

**Table 4:** Descriptive statistics of people living with HIV, preexposure prophylaxis (PrEP) users, postexposure prophylaxis (PEP) users, and people not living with HIV cohorts.

Characteristics		People living with HIV (n=132,664; confidence level 1-2)	People living with HIV (n=152,282; confidence level 1-3)	PrEP users (n=36,088)	PEP users (n=4120)^a^	People not living with HIV (n=20,639,675)
**Race or ethnicity, n (%)**						
	Hispanic or Latinx, any race	20,925 (15.8)	23,346 (15.3)	5829 (16.2)	403 (9.8)	2,628,308 (12.7)
	Non-Hispanic American Indian or Alaska Native	615 (0.5)	684 (0.4)	163 (0.5)	<20 (0.5)	85,524 (0.4)
	Non-Hispanic Asian	2753 (2.1)	3517 (2.3)	1726 (4.8)	222 (5.4)	729,491 (3.5)
	Non-Hispanic Black or African American	49,769 (37.5)	55,144 (36.2)	4534 (12.6)	784 (19)	2,909,443 (14.1)
	Non-Hispanic combined^b^	903 (0.7)	1150 (0.8)	505 (1.4)	40 (1)	281,450 (1.4)
	Non-Hispanic Native Hawaiian or Pacific Islander	247 (0.2)	300 (0.2)	88 (0.2)	<20 (0.5)	67,808 (0.3)
	Non-Hispanic White	48,604 (36.6)	57,479 (37.7)	19,663 (54.5)	2119 (51.4)	11,966,553 (58)
	Unknown	8848 (6.7)	10,662 (7)	3580 (9.9)	512 (12.4)	1,971,098 (9.6)
Age (y), median (IQR)		51 (38-61)	51 (37-61)	36 (30-46)	35 (28-44)	44 (26-63)
**Sex, n (%)**						
	Female	42,093 (31.7)	49,846 (32.7)	4253 (11.8)	2693 (65.4)	11,579,223 (56.1)
	Male	90,496 (68.2)	102,332 (67.2)	31,722 (87.9)	1399 (34)	9,044,853 (43.8)
	Combined or unknown^c^	75 (0.1)	104 (0.1)	113 (0.3)	<20 (0.5)	15,599 (0.1)
**Comorbidity, n (%)**						
	Myocardial infarction	9265 (7)	10,349 (6.8)	482 (1.3)	57 (1.4)	795,829 (3.9)
	Congestive heart failure	10,926 (8.2)	12,106 (7.9)	406 (1.1)	42 (1)	998,313 (4.8)
	Peripheral vascular disease	8002 (6)	8971 (5.9)	352 (1)	52 (1.3)	787,932 (3.8)
	Cerebrovascular disease	9082 (6.8)	10,123 (6.6)	492 (1.4)	76 (1.9)	977,070 (4.7)
	Dementia	3177 (2.4)	3638 (2.4)	225 (0.6)	63 (1.5)	475,742 (2.3)
	Chronic lung disease	40,080 (30.2)	44,328 (29.1)	5617 (15.6)	716 (17.5)	3,562,138 (17.3)
	Rheumatologic disease	10,245 (7.7)	11,502 (7.6)	1280 (3.5)	139 (3.4)	1,097,753 (5.3)
	Peptic ulcer	4445 (3.4)	4919 (3.2)	466 (1.3)	61 (1.5)	335,672 (1.6)
	Liver disease	32,690 (24.6)	35,536 (23.3)	2965 (8.2)	320 (7.8)	1,314,849 (6.4)
	Diabetes	28,307 (21.3)	31,731 (20.8)	2631 (7.3)	321 (7.8)	2,750,693 (13.3)
	Hemiplegia or paraplegia	3751 (2.8)	4124 (2.7)	121 (0.3)	32 (0.8)	281,201 (1.4)
	Kidney disease	26,015 (19.6)	28,288 (18.6)	1142 (3.2)	157 (3.8)	1,681,615 (8.1)
	Cancer	20,035 (15.1)	22,447 (14.7)	1399 (3.9)	143 (3.5)	1,866,452 (9)
	Hypertension	63,025 (47.5)	69,487 (45.6)	7464 (20.7)	691 (16.9)	5,904,993 (28.6)
	Tobacco smoking	34,189 (25.8)	37,120 (24.4)	5830 (16.2)	550 (13.4)	1,844,538 (8.9)
**BMI, n (%)**						
	Underweight	2280 (1.7)	2931 (1.9)	378 (1)	90 (2.2)	1,156,445 (5.6)
	Healthy weight	20,789 (15.7)	23,743 (15.6)	6684 (18.5)	710 (17.3)	2,837,262 (13.7)
	Overweight	35,471 (26.7)	39,718 (26.1)	10,600 (29.4)	864 (21.1)	3,565,102 (17.3)
	Obese	52,872 (39.9)	59,238 (38.9)	12,240 (33.9)	1369 (33.4)	6,260,259 (30.3)
	Missing	21,252 (16)	26,652 (17.5)	6186 (17.1)	1066 (26)	6,820,607 (33)

^a^The PEP cohort is estimated to obfuscate any groups with counts <20 to follow National Clinical Cohort Collaborative governance rules. This will also cause percentages not to sum to 100 in this group.

^b^Includes individuals with race and ethnicity variable values that do not include Hispanic, White, Black or African American, Asian, American Indian or Alaska Native, and Native Hawaiian or Other Pacific Islander.

^c^Includes individuals with sex variable values that do not include male or female.

### HIV-Related Characteristics Among People Living With HIV in N3C

Of the 132,664 people living with HIV in the highest 2 confidence levels, 86,148 (64.9%) had at least 1 VL measurement, and 69,704 (52.5%) had at least 1 CD4 count recorded. The median number of VL and CD4 count laboratory tests was 5 (IQR 2-9) and 4 (IQR 2-8), respectively, per individual (Table 5). Of those with recorded VL and CD4 count measurements, 76,456 (57.6%) had VL and 69,254 (52.2%) had CD4 count results available for analysis (Table 5). Of those who had results, 41,923 (31.6%) had ≥1 detectable VLs (defined as VL >50 copies/mL) and 51,707 (39%) had ≥1 CD4 count <200 cells/µL (Table 5).

**Table 5:** HIV-related characteristics of people living with HIV, preexposure prophylaxis (PrEP) user, postexposure prophylaxis (PEP) user, and people not living with HIV cohorts.

Characteristics			People living with HIV (n=132,664; confidence level 1-2)		People living with HIV (n=152,282; confidence level 1-3)		PrEP users (n=36,088)		PEP users (n=4120)^a^		People not living with HIV (n=20,639,675)
**Individuals with VL^b^ and CD4^c^ measurements, n (%)**											
	≥1 VL measurement	86,148 (64.9)		86,148 (56.6)		3983 (11)		0 (0)		11,390 (0.1)	
	≥1 CD4 measurement	69,704 (52.5)		69,704 (45.8)		259 (0.7)		0 (0)		36,528 (0.2)	
	VL measurements for analysis	76,456 (57.6)		76,456 (50.2)		3483 (9.7)		0 (0)		7599 (0)	
	CD4 measurement for analysis	69,254 (52.2)		69,254 (45.5)		238 (0.7)		0 (0)		36,121 (0.2)	
**VL and CD4 measurements per person (n), median (IQR)**											
	VL measurement count	5 (2-9)		5 (2-9)		1 (1-2)		0 (0-0)		1 (1-1)	
	CD4 measurement count	4 (2-8)		4 (2-8)		1 (1-1)		0 (0-0)		1 (1-3)	
**Individuals per VL category (copies/mL), n (%)**											
	VL ≥50	41,923 (31.6)		41,923 (27.5)		0 (0)		0 (0)		0 (0)	
	VL <50	34,533 (26)		34,533 (22.7)		3483 (9.7)		0 (0)		7599 (0)	
	VL unknown	9692 (7.3)		9692 (6.4)		500 (1.4)		4120^a^ (100)		3792 (0)	
	VL null	46,516 (35.1)		66,134 (43.4)		32,105 (89)		0 (0)		20,628,284 (99.9)	
**Individuals per CD4 category (cells/μL), n (%)**											
	CD4 >200	51,707 (39)		51,707 (34)		238 (0.7)		0 (0)		27,718 (0.1)	
	CD4 ≤200	17,547 (13.2)		17,547 (11.5)		0 (0)		0 (0)		8403 (0)	
	CD4 unknown	450 (0.3)		450 (0.3)		21 (0.1)		4120^a^ (100)		407 (0)	
	CD4 null	62,960 (47.5)		82,578 (54.2)		35,829 (99.3)		0 (0)		20,603,147 (99.8)	
Individuals with HIV-related conditions and negative laboratory results, n (%)			11,048 (8.3)		11,048 (7.3)		0 (0)		0 (0)		0 (0)

^a^The PEP cohort is estimated to obfuscate any groups with counts <20 to follow National Clinical Cohort Collaborative governance rules.

^b^VL: viral load.

^c^CD4: cluster of differentiation 4.

### COVID-19 Outcomes in People Living With HIV, PrEP Users, and PEP Users in N3C

We examined the distribution of COVID-19–related variables within the cohorts of people living with HIV, PrEP users, and PEP users in comparison to people not living with HIV. The number of individuals who were tested for COVID-19, regardless of the results, was 104,903 (79.1%) among 132,664 people living with HIV (confidence levels 1 and 2), 30,799 (85.3%) for 36,008 PrEP users, 3704 (89.9%) for 4120 PEP users, and 17,756,497 (86%) in 20,639,675 people not living with HIV (Table 6). The proportions who tested positive for COVID-19 were similar between the cohorts; there were 34.4% (45,609/132,664) people living with HIV, 33.6% (12,121/36,088) PrEP users, 37% (1525/4120) PEP users, and 38.6% (7,970,336/20,639,675) people not living with HIV who tested positive for COVID-19 (Table 6). When categorizing individuals based on the severity of COVID-19, a higher proportion of people living with HIV experienced a COVID-19–related hospitalization (4650/132,664, 3.5%), COVID-19–related mortality (828/132,664, 0.6%), and all-cause mortality (2083/132,664, 1.6%) outcomes compared to people not living with HIV who had a lower proportion of COVID-19–related hospitalization (407,894/20,639,675, 2%), COVID-19–related mortality (95,421/20,639,675, 0.5%), and all-cause mortality (192,519/20,639,675, 0.9%; Table 6). With regard to receipt of COVID-19 vaccinations, people living with HIV exhibited a lower proportion of full primary (ie, completing 2 doses when applicable) vaccination documentation (15,387/132,664, 11.6%) compared to PrEP users (5414/36,088, 15%), although it was higher compared to PEP users (441/4120, 10.7%) and similar to people not living with HIV (2,327,522/20,639,675, 11.3%; Table 6). The booster vaccination documentation displayed a lower proportion of people living with HIV receiving any booster vaccination (28,520/132,664, 21.5%) compared to PrEP users (12,547/36,088, 34.8%) but higher than PEP users (559/4120, 13.6%) and people not living with HIV (2,521,638/20,639,675, 12.2%; Table 6).

**Table 6:** COVID-19–related characteristics of the people living with HIV, preexposure prophylaxis (PrEP) user, postexposure prophylaxis (PEP) user, and people not living with HIV cohorts.

Characteristics			People living with HIV (n=132,664; confidence level 1-2), n (%)		People living with HIV (n=152,282; confidence level 1-3), n (%)		PrEP users (n=36,088), n (%)		PEP users (n=4120)^a^, n (%)		People not living with HIV (n=20,639,675), n (%)
**COVID-19 testing and outcomes**											
	COVID-19 screening	104,903 (79.1)		121,130 (79.5)		30,799 (85.3)		3704 (90.4)		17,756,497 (86)	
	COVID-19 positivity	45,609 (34.4)		52,223 (34.3)		12,121 (33.6)		1525 (37.2)		7,970,336 (38.6)	
	COVID-19–associated hospitalization	4650 (3.5)		5259 (3.5)		226 (0.6)		51 (1.2)		407,894 (2)	
	COVID-19–associated mortality	828 (0.6)		1027 (0.7)		<20 (0.1)		<20 (0.5)		95,421 (0.5)	
	All-cause mortality among individuals with COVID-19	2083 (1.6)		2412 (1.6)		40 (0.1)		<20 (0.5)		192,519 (0.9)	
	Had ≥1 COVID-19 reinfection	2864 (2.2)		3204 (2.1)		612 (1.7)		118 (2.9)		353,177 (1.7)	
**COVID-19 vaccination**											
	No vaccination documented	81,096 (61.1)		95,300 (62.6)		15,855 (43.9)		2922 (71.3)		15,087,131 (73.1)	
	Partial primary vaccination series documented	7661 (5.8)		8464 (5.6)		2272 (6.3)		177 (4.3)		703,384 (3.4)	
	Full primary vaccination series documented	15,387 (11.6)		17,301 (11.4)		5414 (15)		441 (10.7)		2,327,522 (11.3)	
	Booster vaccination documented	28,520 (21.5)		31,217 (20.5)		12,547 (34.8)		559 (13.6)		2,521,638 (12.2)	

^a^The PEP cohort is estimated to obfuscate any groups with counts <20 to follow National Clinical Cohort Collaborative governance rules.

## Discussion

### Principal Findings

Using an extensive phenotyping algorithm that leverages granular data for medical conditions, laboratory measurements, and drug exposures available in an EHR repository, we have identified people living with HIV, PrEP users, and PEP users, augmented by confidence levels to provide flexibility in cohort selection to address potential misclassification. Our algorithm was refined using an iterative process with multiple reviews by clinicians with HIV experience to reduce misclassification. Our work allows for rapid identification of people living with HIV in large datasets originating from multiple health systems, with reduced misclassification among PrEP or PEP users, those with HBV infection only, or those prescribed ritonavir only for COVID-19 treatment. Our computational phenotyping approach allows for greater transferability to other EHR data sources, accounts for nuances after the emergence of COVID-19, and is applicable for optimizing future research. Our approach lays the groundwork for large epidemiological investigations among people living with HIV and those at risk of acquiring HIV [13,14,38-41], noting that analyses to date that use this phenotyping have influenced guidance for COVID-19 vaccination prioritization for people living with HIV and can inform interventions, ranging from addressing clinical to social needs.

We have identified one of the largest cohorts of people living with HIV in the United States, leveraging a nationally sampled EHR repository. The overall distribution of sociodemographic, comorbidity, and COVID-19 findings of our cohorts is consistent with available literature. We identified a higher proportion of diabetes, hypertension, and chronic lung disease in people living with HIV than in people not living with HIV, which reflects the epidemiology of comorbidity burden for HIV in the United States [1,42]. Similarly, where laboratory results were available, our proportion of people living with HIV with undetectable VL (34,533/132,664, 26%) reflects the HIV care continuum for the United States [1]. In addition, the demographics for PrEP users are also consistent with what has been previously reported in the United States. Similarly, with regard to COVID-19, we saw increased COVID-19–related hospitalizations, mortality, and all-cause mortality in people living with HIV than in people not living with HIV, which has been previously reported in other cohorts of people living with HIV [43-46]. Nonetheless, appreciating the nuanced similarities and differences between our N3C cohorts and others helps articulate places of generalizability to the HIV or PrEP populations in the United States.

One key element of our computational phenotype, with clinician annotation, aimed to minimize potential misclassification. For instance, in the entire N3C, we identified 121,099 individuals with a condition for HIV, but 16,185 (13.4%) of them had tested negative for HIV, suggesting these individuals may be misclassified if we used medical conditions alone. In our experience, it also appeared that medical conditions were occasionally applied inappropriately for those who were being screened for HIV or receiving prescriptions for PrEP or PEP. Many of the published HIV case-finding algorithms, with specificity frequently >90%, were validated before approval of and more expanded access to PrEP or nonoccupational PEP [47,48]. Thus, the potential for misclassification is greater now than ever.

Our computational phenotyping approach parallels other recent work, although arguably, it adds nuances that help reduce potential misclassification. Yang et al [23] conducted a study involving computational phenotyping of people living with HIV and PrEP users within the All of Us research program. They used a hybrid approach, with both EHR and self-reported survey data, to identify people living with HIV. Notably, their findings indicated that the identification of people living with HIV was most prevalent through a combination of drug exposures and medical conditions (n=3324 individuals, constituting 43.4% as opposed to our study with 14,595/152,282, 9.6%) as well as drug exposure alone (n=2191 individuals, accounting for 28.6% as opposed to our study with 11,510/152,282, 7.6%), with 19.9% (43,419/216,971) of the individuals with drug exposure only to ritonavir [23]. Similarly, in the HIV-Phen algorithm, May et al [24] identified misclassification with *ICD-10* codes alone, including roughly 6.5% of individuals who only had a screening test. Within the Veterans Health Administration, using a single *ICD-10* code alone provided a positive predictive value of only 69% [25]. Notably, among our cohort of people not living with HIV, 6065 individuals had inappropriate HIV-related conditions assigned despite their data suggesting they are not living with HIV, and 4159 (68.6%) of them had only a single medical condition; 58 of the PrEP users in our PrEP cohort had a condition for HIV inappropriately assigned. This misclassification was relatively small for the people not living with HIV cohort (6065/20,639,675, 0.03%); however, it would make up 4% of our people living with HIV cohort if they were included. This underscores the fallibility of relying on diagnostic codes alone, and our work can provide estimates of misclassification for researchers who may not have access to additional clinical data (ie, those limited to claims data). To help address the low specificity of medical conditions alone, we used a comprehensive approach evaluating not only positive or negative laboratory results to identify our cohorts but also repeated laboratory measurements over time. For example, an instance of an individual with one row of medical condition data followed by multiple negative HIV antigen or antibody tests is more consistent with an HIV-related condition assigned incorrectly. In contrast, an instance of an individual with one row of medical condition data followed by repeated VL and CD4 laboratory measurements is more consistent with an HIV-related condition being assigned correctly. We estimate that using medical conditions alone in other published algorithms, approximately 13.4% of probable people living with HIV were potentially misclassified according to the methods developed by Yang et al [23]. Adding an algorithm to identify PEP users also allows for reduced misclassification, as these individuals were identified by drug exposures only and would be classified as people living with HIV if not properly removed.

Another novel element of our computational phenotyping is the ability to identify individuals at risk for HIV through both PrEP and PEP exposures. Unfortunately, due to low numbers of people with exposure to long-acting injectables (cabotegravir and lenacapavir) in N3C, these were not included in our phenotyping; however, this work is currently in progress as the use of these agents expands. Among the aforementioned studies, only one [23] conducts computational phenotyping to identify PrEP or PEP users, with the latter being a group of people exposed to HIV drugs but only for a short time and, thus, embodying an element of temporality that is only available in longitudinal datasets. This provides the benefit of reducing potential misclassification between these 3 cohorts with antiretroviral drug exposure for differing reasons and provides additional comparator groups at risk of acquiring HIV for analyses. Nonetheless, readers should consider that our overall approach lacks source validation, as N3C prohibits reidentification of individuals with site data. While our clinician annotation provided measures of high consistency, we are unable to assess accuracy. Thus, we are limited in adjudicating further these reductions in potential misclassification without source validation.

With the emergence of COVID-19 and one of its primary treatments (ie, oral nirmatrelvir/ritonavir), which includes an antiretroviral also used for HIV treatment, using a single antiretroviral alone after COVID-19 can result in misclassification. Out of the entire N3C population, we identified 0.21% (43,149/20,832,144) of the individuals with only ritonavir exposure and 0.03% (6586/20,832,144) with ritonavir exposure and an HIV laboratory measurement. From this point forward, in the post–COVID-19 era, additional steps need to be implemented to reduce misclassification of people not living with HIV as people living with HIV when isolated ritonavir exposures exist. Yang et al [23] identified 2191 individuals (accounting for 28.6% of people living with HIV) by drug exposure alone; 19.9% (43,149/216,971) of our study’s drug-only population had ritonavir-only exposures, underscoring the point that, after the emergence of COVID-19, potential misclassification based on drug alone may be a significant issue [23].

A clinician-annotated approach to computational phenotyping of a cohort involving individuals with HIV is desirable from several points of view, engendering, for example, the use of clinically relevant data and insights. However, such efforts are less reproducible and repeatable over time. Thus, future work in automation, for example, through machine learning, could provide unique insights into how such a cohort can more readily be derived in any EHR dataset. Future research can assess various performance characteristics of clinician-annotated or curated approaches to automation approaches, as we acknowledge that our clinician-annotated approach was time consuming and may face challenges in transferability to other EHR repositories, despite our best efforts in making our phenotyping approach and code as transparent as possible.

### Strengths and Limitations

While our work has several strengths, including identifying one of the largest cohorts of people living with HIV in the United States, demonstrating high consistency with our phenotyping using clinician annotation, and identifying PrEP and PEP users, it also faces several challenges. First, as already noted, the lack of source validation or any external validation limits our ability to assess the accuracy of the phenotypes. That being said, given our use of highly specific HIV laboratory data and repeated measures over time, we are extremely confident in our identification of people living with HIV in our confidence levels 1 and 2. Second, our dataset contains a significant amount of noninformative data. For example, there are instances where we can ascertain that an HIV screening test was performed, but the result is not available in an interpretable form, or a CD4 count is available, but the units are missing. This is likely a by-product of data ingestion and harmonization across multiple different health systems; while major strides have been made in EHR interoperability, more work needs to be done to meaningfully use all available data in the various EHRs and improve the quality of data ingestion processes. Third, while the N3C Enclave cohort is inclusive of multiple health systems across the United States, it is still only inclusive of individuals with encounters with the health care system, hence various characteristics, including COVID-19 outcomes, may not be fully generalizable to those affected by HIV with limited or no access to care, who are often the most vulnerable of the vulnerable.

### Conclusions

Using an extensive phenotyping algorithm leveraging granular data in an EHR repository for medical conditions, laboratory measurements, and drug exposures, we have identified people living with HIV, PrEP users, and PEP users with high precision in the post–COVID-19 era. Our approach offers flexibility to select cohorts with confidence levels that best fit the needs of the research question under investigation, with regard to potential misclassification. We offer transferable lessons to optimize future EHR phenotyping for these cohorts. Our approach lays the groundwork for large epidemiological investigations among people living with HIV and those at risk for acquiring HIV and can inform interventions addressing clinical and social needs.

## Appendix

Multimedia Appendix 1 Supplementary methods for applying confidence levels to people living with HIV, pre-exposure prophylaxis users, the clinician annotation activity, demographic and comorbidity definitions, and COVID-19 outcomes definitions.

Multimedia Appendix 2 Performance of our clinician annotation activity in people living with HIV, pre-exposure prophylaxis, postexposure prophylaxis, and people not living with HIV cohorts.

## Abbreviations

CD4: cluster of differentiation 4
EHR: electronic health record
HBV: hepatitis B virus
ICD-10: International Classification of Diseases, Tenth Revision
IRB: Institutional Review Board
N3C: National Clinical Cohort Collaborative
NIH: National Institutes of Health
OMOP: Observational Medical Outcomes Partnership
PEP: postexposure prophylaxis
PrEP: preexposure prophylaxis
SNOMED CT: Systemized Medical Nomenclature for Medical Clinical Terminology
TDF: tenofovir disoproxil fumarate
VL: viral load

## Data Availability

The National Clinical Cohort Collaborative (N3C) data transfer to the National Center for Advancing Translational Sciences is performed under a Johns Hopkins University Reliance Protocol (IRB00249128) or individual site agreements with the National Institutes of Health (NIH). The N3C Data Enclave is managed under the authority of the NIH; information can be found [54]. N3C Enclave data are protected and can be accessed for COVID-19–related research with an NIH-approved (1) IRB protocol and (2) institutional Data Use Request (DUR ID: RP-CA3365). A detailed accounting of data protections and access tiers is found at the NCATS website [54]. N3C Enclave and data access instructions can be found at the N3C website [55]; all codes used to produce the analyses in this manuscript are available within the N3C Enclave to users with valid log-in credentials to support reproducibility.
