# Supplementary material for: Identifying People Living With or Those at Risk for HIV in a Nationally Sampled Electronic Health Record Repository Called the National Clinical Cohort Collaborative: Computational Phenotyping Study
Source: JMIR Med Inform. 2025 Jul 11;13:e68143. doi: 10.2196/68143 (PMC12299939; doi:10.2196/68143)
Supplement: Multimedia Appendix 1 [file medinform_v13i1e68143_app1.doc]

# Supplementary Methods

## Applying levels of confidence to the cohort of people living with HIV (PLWH)

Each confidence level constituted one or more criteria, with a total of 12 hierarchical, mutually-exclusive criteria across the three confidence levels based on conditions, laboratory measurements, and drug exposure data tables akin to similar work in other disease areas.[^1,2^](https://www.zotero.org/google-docs/?DA1ldZ) Confidence level 1 mainly consisted of individuals with “positive” HIV-related laboratory tests, such as VL measurement >50 copies/mL, a positive HIV Antibody/Antigen (Ag/Ab) test, or enzyme-linked immunosorbent assay (ELISA). Confidence level 2 PLWH centered around individuals identified by a combination of conditions, laboratory measurements, and/or antiretroviral (ARV) drug exposures with records of laboratory measurements but no available results. Lastly, confidence level 3 PLWH mainly consisted of individuals identified by drugs only or only 1 record of a HIV diagnosis. We consider confidence levels 1 and 2 to be high and level 3 as medium.

## Assigning confidence levels for PrEP users

PrEP users were evaluated for their confidence level using a scale from 1 to 3, where 1 indicated the highest level of confidence for each observation available for an individual. Ultimately, each individual was assigned a final confidence level, determined by selecting the highest confidence level (i.e., most confident) among the rows associated with that individual. Individuals were placed into confidence level 1 if they had a confirmed negative HIV-related screening laboratory result (e.g., an HIV Ag/Ab test, ELISA, etc.). For level 2, individuals were identified as a PrEP user by drugs with records of HIV-related screening laboratory results but without a confirmatory negative result. Level 3 PrEP users were identified by drugs and did not have any record of HIV-related laboratory results.

## Clinician annotation activity

A total of 120 (0.0006%) individuals were randomly selected, with 90 (0.07%) from the HIV positive cohort (10 individuals from each possible combination of CD4 and VL measurements, categorized by CD4 counts of ≤200 cells/uL, >200 cells/uL, or null/missing and VL measurements of ≥50 copies/mL, <50 copies/mL, or null/missing), 10 (0.03%) from the PrEP cohort, 10 (0.24%) from the PEP cohort, and 10 (0.00005%) from the HIV negative cohort. Three clinicians with experience in HIV were provided all available HIV-related EHR data for these individuals inside the N3C Enclave (i.e., conditions, laboratory measurements, and drugs) and tasked with classifying them as PLWH, PrEP, PEP users, or PNLWH, while blinded to the relative proportions and phenotyping algorithms’ classification (noting, these clinicians knew the overall approach of our phenotyping algorithm but not specifics). Criteria were modified based on identified patterns of likely misclassification, and annotation was repeated through an iterative process until we no longer had individuals with an unclear or inappropriate designation. For example, we identified multiple individuals within the PLWH cohort who had an HIV-specific condition, followed by a subsequent negative HIV Ag/Ab screening test, indicating a condition was likely inappropriately assigned in the setting of screening for HIV. We adjusted our criteria and confirmed appropriate classification with repeated annotation. We subsequently evaluated how individuals were categorized (i.e., PLWH, PrEP users, PEP users, or PNLWH) by our computational phenotyping compared to classifications made by the HIV clinicians. To assess the accuracy of our phenotyping, we measured levels of precision, recall, and an F1-score when comparing the "predicted" phenotypes generated by our algorithm to the "actual" phenotypes determined by our clinicians. Additionally, we conducted a comparison of clinician assessments using Cohen's kappa test to assess inter-rater agreement.

## Demographic and Comorbidity Definitions

We combined race and ethnicity variables into the following groups: non-Hispanic (NH)-Black, Hispanic/Latinx of any race, NH-Asian American, Native Hawaiian or Pacific Islander (AANHPI), NH-American Indian or Alaskan Native (NH-AIAN), NH-White and unknown/missing. Sex was classified as female, male or unknown/missing. For those with a COVID-19 diagnosis, we used age at the time of COVID-19 diagnosis as the index date. In those without a COVID-19 diagnosis, date of birth and the date of data access from N3C was used to calculate age for the equivalent of an “index” date. We defined comorbidities including hypertension, chronic kidney disease, diabetes mellitus, and Hepatitis C by OMOP concept IDs. We defined body mass index (BMI) data for each individual using the maximum value based on height and weight measurements and categorized BMI using guidelines set by the Centers for Disease Control and Prevention (CDC).[^3^](https://www.zotero.org/google-docs/?t9wsbt)

## COVID-19 outcomes definitions

We assessed COVID-19 severity by analyzing the number of individuals who experienced COVID-19 related hospitalization (a binary outcome of whether the patient was hospitalized in the day prior to up to 16 days after the COVID-19 diagnosis date), COVID-19 related mortality, (a binary column indicating an individual died within 60 days following the COVID-19 index date), or all-cause mortality (a binary outcome of whether the patient experienced death or discharge to hospice over the entire period of their observation time available in N3C).[^4^](https://www.zotero.org/google-docs/?kv4a53) In regards to COVID-19 vaccination, we ascertained documentation of partial initial (defined as only one dose of the two mRNA vaccine doses), full initial (defined as two doses of the two mRNA vaccines or one dose of the Janssen vaccine), or any booster (defined as any COVID-19 vaccination after full initial vaccination) vaccination.

**References:**

[1. Jones SE, Bradwell KR, Chan LE, et al. Who is pregnant? Defining real-world data-based pregnancy episodes in the National COVID Cohort Collaborative (N3C). *JAMIA Open*. 2023;6(3):ooad067. doi:10.1093/jamiaopen/ooad067](https://www.zotero.org/google-docs/?KjjBl0)

[2. Development and Evaluation of a Standardized Research Definition for Opioid Overdose Outcomes - Ingrid A. Binswanger, Komal J. Narwaney, Edward M. Gardner, Barbara A. Gabella, Susan L. Calcaterra, Jason M. Glanz, 2019. Accessed September 6, 2024. https://journals.sagepub.com/doi/10.1080/08897077.2018.1546263](https://www.zotero.org/google-docs/?KjjBl0)

[3. CDC. All About Adult BMI. Centers for Disease Control and Prevention. June 3, 2022. Accessed May 23, 2024. https://www.cdc.gov/healthyweight/assessing/bmi/adult_bmi/index.html](https://www.zotero.org/google-docs/?KjjBl0)

[4. N3C. National COVID Cohort Collaborative. GitHub. Accessed May 23, 2024. https://github.com/National-COVID-Cohort-Collaborative](https://www.zotero.org/google-docs/?KjjBl0)
