# Supplementary material for: Identifying People Living With or Those at Risk for HIV in a Nationally Sampled Electronic Health Record Repository Called the National Clinical Cohort Collaborative: Computational Phenotyping Study
Source: JMIR Med Inform. 2025 Jul 11;13:e68143. doi: 10.2196/68143 (PMC12299939; doi:10.2196/68143)
Supplement: Multimedia Appendix 2 [file medinform_v13i1e68143_app2.doc]

**Table S1: Performance of our clinician annotation activity in PLWH, PrEP, PEP, and PNLWH cohorts.**

| **Confusion matrix** | | | | | |
| --- | --- | --- | --- | --- | --- |
|  |  | Algorithm reference | | | |
|  | Clinician predicted | HIV positive (conf 1-3) | PrEP | PEP | HIV Negative |
| Clinician 1 | HIV positive (conf 1-3) | 86 | 1 | 0 | 0 |
| Clinician 1 | PrEP | 1 | 0 | 0 | 10 |
| Clinician 1 | PEP | 3 | 0 | 10 | 0 |
| Clinician 1 | HIV Negative | 0 | 9 | 0 | 0 |
| Clinician 2 | HIV positive (conf 1-3) | 87 | 0 | 0 | 0 |
| Clinician 2 | PrEP | 0 | 0 | 0 | 10 |
| Clinician 2 | PEP | 3 | 0 | 10 | 0 |
| Clinician 2 | HIV Negative | 0 | 10 | 0 | 0 |
| Clinician 3 | HIV positive (conf 1-3) | 87 | 1 | 0 | 0 |
| Clinician 3 | PrEP | 0 | 0 | 0 | 10 |
| Clinician 3 | PEP | 3 | 0 | 10 | 0 |
| Clinician 3 | HIV Negative | 0 | 9 | 0 | 0 |
|  |  |  |  |  |  |
| **Performance metrics** | | | | | |
| Person | Metric | HIV positive (conf 1-3) | PrEP | PEP | HIV Negative |
| Clinician 1 | Sensitivity | 0.96 | 1.00 | 1.00 | 0.90 |
| Clinician 1 | Specificity | 0.97 | 0.99 | 0.97 | 1.00 |
| Clinician 1 | Positive predictive value | 0.99 | 0.91 | 0.77 | 1.00 |
| Clinician 1 | Negative predictive value | 0.88 | 1.00 | 1.00 | 0.99 |
| Clinician 1 | Precision | 0.99 | 1.00 | 0.77 | 0.91 |
| Clinician 1 | Recall | 0.96 | 0.90 | 1.00 | 1.00 |
| Clinician 1 | F1-score | 0.97 | 0.95 | 0.87 | 0.95 |
| Clinician 2 | Sensitivity | 0.97 | 1.00 | 1.00 | 1.00 |
| Clinician 2 | Specificity | 1.00 | 1.00 | 0.97 | 1.00 |
| Clinician 2 | Positive predictive value | 1.00 | 1.00 | 0.77 | 1.00 |
| Clinician 2 | Negative predictive value | 0.91 | 1.00 | 1.00 | 1.00 |
| Clinician 2 | Precision | 1.00 | 1.00 | 0.77 | 1.00 |
| Clinician 2 | Recall | 0.97 | 1.00 | 1.00 | 1.00 |
| Clinician 2 | F1-score | 0.98 | 1.00 | 0.87 | 1.00 |
| Clinician 3 | Sensitivity | 0.97 | 1.00 | 1.00 | 0.90 |
| Clinician 3 | Specificity | 0.97 | 1.00 | 0.97 | 1.00 |
| Clinician 3 | Positive predictive value | 0.99 | 1.00 | 0.77 | 1.00 |
| Clinician 3 | Negative predictive value | 0.91 | 1.00 | 1.00 | 0.99 |
| Clinician 3 | Precision | 0.99 | 1.00 | 0.77 | 1.00 |
| Clinician 3 | Recall | 0.97 | 0.90 | 1.00 | 1.00 |
| Clinician 3 | F1-score | 0.98 | 0.95 | 0.87 | 1.00 |
|  |  |  |  |  |  |
| **Inter-rater reliability** | | | | | |
|  | Clinician 1 | Clinician 2 | Clinician 3 |  |  |
| Clinician 1 | 1.00 | 0.93 | 0.91 |  |  |
| Clinician 2 |  | 1.00 | 0.98 |  |  |
| Clinician 3 |  |  | 1.00 |  |  |
